# PEMWE with Internal Real-Time Microscopic Monitoring Function

**DOI:** 10.3390/membranes11020092

**Published:** 2021-01-27

**Authors:** Chi-Yuan Lee, Chia-Hung Chen, Guo-Bin Jung, Yu-Xiang Zheng, Yi-Cheng Liu

**Affiliations:** 1 Department of Mechanical Engineering, Yuan Ze Fuel Cell Center, Yuan Ze University, Taoyuan 32003, Taiwan; guobin@saturn.yzu.edu.tw (G.-B.J.); f129771057f@gmail.com (Y.-X.Z.); pierce0922@gmail.com (Y.-C.L.); 2 HOMYTECH Global Co., Ltd., Taoyuan 33464, Taiwan; chenjahon@gmail.com

**Keywords:** PEMWE, MEMS, flexible six-in-one microsensor

## Abstract

In recent years, various countries have been paying attention to environmental protection issues, believing that climate change is the main challenge to the developed countries’ energy policies. The most discussed solution is renewable energy. The energy storage system can reduce the burden of the overall power system of renewable energy. The hydrogen energy is one of the optimal energy storage system options of renewable energy at present. According to these policies and the future trend, this study used micro-electro-mechanical systems (MEMS) technology to integrate micro voltage, current, temperature, humidity, flow and pressure sensors on a 50 μm thick polyimide (PI) substrate. After the optimization design and process optimization, the flexible six-in-one microsensor was embedded in the proton exchange membrane water electrolyzer (PEMWE) for internal real-time microscopic monitoring.

## 1. Introduction

Since the industrial revolution, the large amount of CO_2_ emission has aggravated the global greenhouse effect gradually [1,2,3]. The global warming is recognized as the consequence of the large consumption of fossil fuels (e.g., coal, petroleum and natural gas); it will cause major disasters, such as flood disaster flood and drought [4,5,6]. At present, in order to solve this increasingly serious problem, many advanced countries are making efforts and investing resources [7]. The Paris Agreement was adopted at the COP 21 in 2015 to limit global temperature rise to 1.5 °C [8]. The Korean government declared in 2019 that the greenhouse gas emission in 2017 was reduced by 709.1 million tons, and it would be reduced by 536 million tons in 2030, so as to achieve a sustainable low-carbon green society [9,10]. In 2020, the German government issued “Energiewende”, changing the energy mode from fossil fuels into renewable energy. In comparison to the greenhouse gas emission in 1990, the primary objective is to reduce the greenhouse gas emission by 40% in 2020, by 55% in 2030, by 70% in 2040, and by 95% in 2050 [11,12].

In recent years, various countries are paying attention to environmental issues, believing that climate change is the main challenge to the energy policies of developed countries. The current target is to use renewable natural energy resource flows to provide power for society instead of the energy reserves, which are about to be exhausted. However, the solar energy or wind energy has a fatal problem, i.e., unstable power supply, so the energy storage device will play a leading part. Among the present energy storage devices, the extensively used lithium battery has several problems, the lithium used in the lithium battery is a scarce resource, large consumption will result in high cost, it is not a suitable large energy storage device, and the leakage of electrolyte may cause burning or even explosion. The hydrogen energy storage device is one of the optimal energy storage systems of green energy [13].

Chakik et al. [14] tried different voltage conditions to find out the optimal condition for making hydrogen. Trinke et al. [15] tested the influence of the current density on the oxygen flux in the cathode outlet with and without Pt catalyst. Müller et al. [16] studied different temperatures (60, 70, 80 and 90 °C) and drag coefficients (1.0, 1.5, 2.0, 2.5) and the transmission relationship between Nafion membrane thickness and water spreading. Sartory et al. [17] operated water electrolyzer under different working conditions, the result showed that the higher the temperature was, the higher was the hydrogen production efficiency. Fujimura et al. [18] studied the influence of surface wettability on hydrogen evolution reaction (HER) activity and efficiency. Saccardo et al. [19] used different cations to adhere to a Nafion117-MEA membrane material surface; the experiment proved that the humidity could influence the performance of the membrane electrode assembly (MEA) membrane material. Möckl et al. [20] built an internal heat transfer model of a polymer electrolyte membrane water electrolyzer (PEMWE), and performed experiments on heat transfer and flow control. The experiments proved that the flow control could effectively control the temperature difference between the runner inlet and outlet in a specific range, preventing MEA from being damaged by overheating. Ferrero et al. [21] studied the performance differences of PEM water electrolyzer in different water flows, and found that high pressure resulted in higher performance, and low pressure resulted in lower performance. Grigoriev et al. [22] studied the operation of PEMWE at a constant temperature of 85 °C and different pressures, and found that increasing the pressure appropriately could reduce the energy consumption of electrolyzed water effectively.

Responding to the trend of these policies and the bottleneck of internal diagnosis of PEMWE, this study used MEMS technology to integrate micro-voltage, current, temperature, humidity, flow and pressure sensors on a 50 μm thick PI substrate, after optimization design and process optimization, the flexible six-in-one microsensor was embedded in the PEMWE for internal real-time microscopic monitoring.

## 2. Sensing Principle of Flexible Six-in-One Microsensor

### 2.1. Micro Voltage Sensor

**Figure 1:**
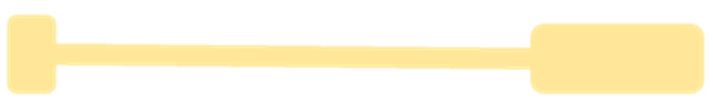
Schematic diagram of micro voltage sensor.

This study used a microminiaturized metal probe as a micro voltage sensor. In this study, only the sensing area of the micro voltage sensor and the tail end linked with the circuit board are exposed, the rest is insulated by a PI 9320 insulating layer, so as to detect the voltage in local region, as shown in Figure 1.

### 2.2. Micro Current Sensor

**Figure 2:**
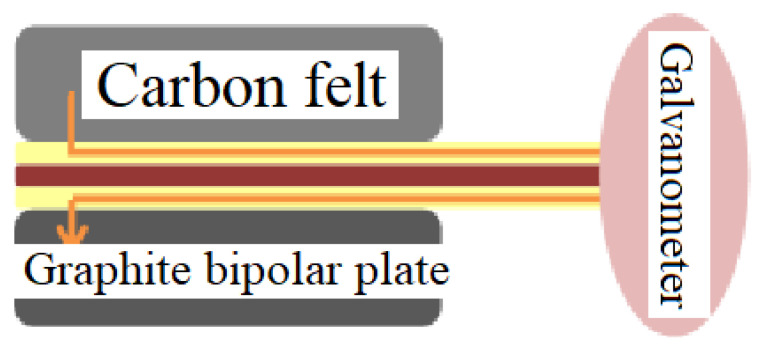
Schematic diagram of operating principle of micro current sensor.

This study uses a microminiaturized metal probe as the micro current sensor using the same principle as the micro voltage sensor, only detecting the current in a local region, as shown in Figure 2.

### 2.3. Micro Temperature Sensor

**Figure 3:**
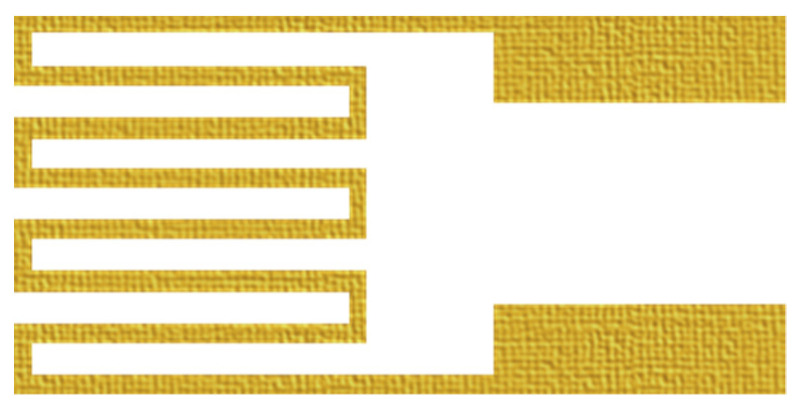
Schematic diagram of resistive micro temperature sensor.

This study used the RTD sensing principle. In a certain temperature range, the resistance of metal varies with temperature. The sensor structure is shown in Figure 3. The Au is selected as the material of RTD for stable chemical properties, simple process and high linearity. The relation can be reduced to Equation (1).

*R_t_* ≈ *R*_0_(1 + *α* × Δ*T*) (1)

### 2.4. Micro Humidity Sensor

**Figure 4:**
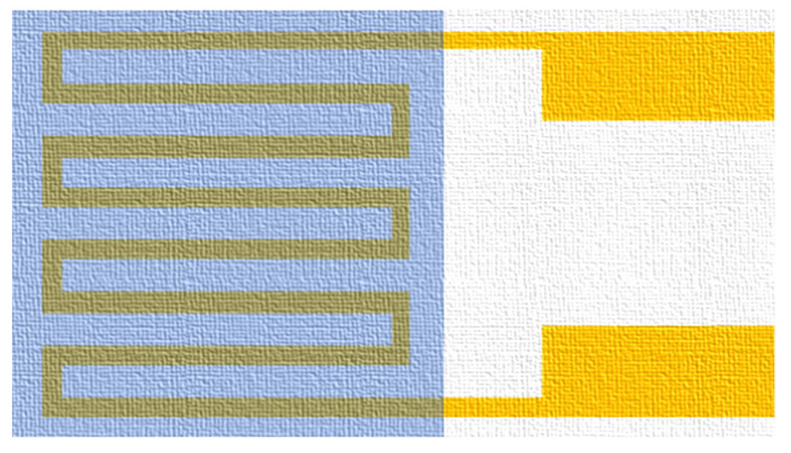
Schematic diagram of resistive micro humidity sensor.

The micro humidity sensor uses PI 9305 as the humidity sensing material in this study. Generally, this material must have a nonconducting property, when the volume of material increases with moisture pickup, the adhering circuit resistance increases too, as shown in Figure 4.

### 2.5. Micro Flow Sensor

**Figure 5:**
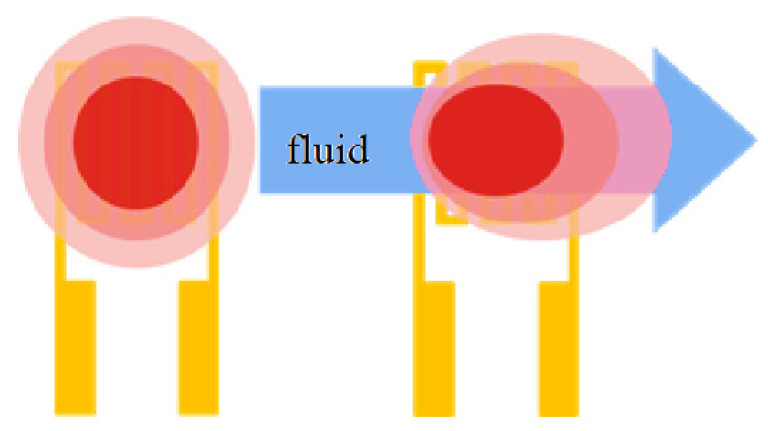
Schematic diagram of principle of hot-wire micro flow sensor.

In terms of the principle of a hot-wire micro flow sensor, the resistance heater generates a heat source with a constant voltage input and as the heat carried away by the fluid flow increases, the resistivity of the resistance heater decreases. The principle of the hot-wire micro flow sensor is shown in Figure 5. According to the King’s law, the relation between the heat dissipation rate and the fluid flow rate is expressed as Equation (2), when the flow is not zero, Equation (2) can be changed to Equation (3).

*Q* = *I*^2^ × *R* = *I* × *V* = (*A* + *B* × *U^n^*) (*T_s_* − *T_o_*) (2)

*Q* = (*A* + *B* × *U*^0.5^) Δ*T* (3)

### 2.6. Micro Pressure Sensor

In terms of the general capacitive pressure sensor, a dielectric layer of non-conducting material is sandwiched in between two parallel electrodes, forming a sandwich structure. The computing equation of the capacitance value between two parallel electrodes is expressed as Equation (4).

(4) $$\Delta C=\varepsilon_{r}\varepsilon_{0}\frac{A}{\Delta d}$$

where *ε*_0_ is constant 8.854 × 10^−12^ (F/m), *ε_r_* is the dielectric constant of material, *A* is the projection overlapping area of two parallel electrodes, Δ*d* is the rate of change in distance between two electrodes.

**Figure 6:**
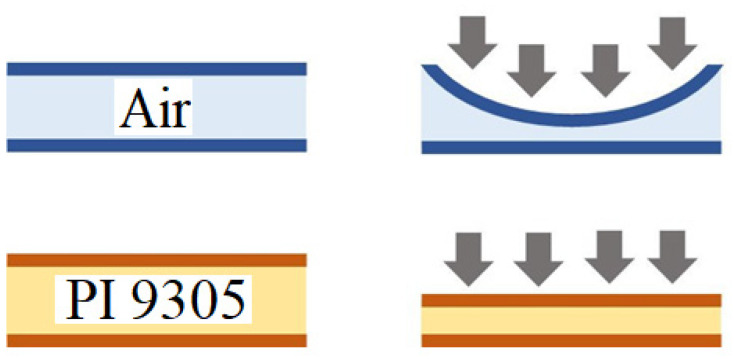
Dielectric layer deformation mode.

The commercially available pressure sensor is a hollow structure, when it receives pressure, the thin film deformation is nonlinear, inducing poor sensor linearity and sensitivity. In order to solve the above problems, Fujifilm Durimide^®^ PI 9305 with high dielectric constant and small E-modulus is selected as dielectric layer in design, the solid dielectric layer can be stressed and deformed evenly, matching the linear Equation (4), the mode of deformation is shown in Figure 6.

## 3. Process Development of Flexible Six-in-One Microsensor

**Figure 7:**
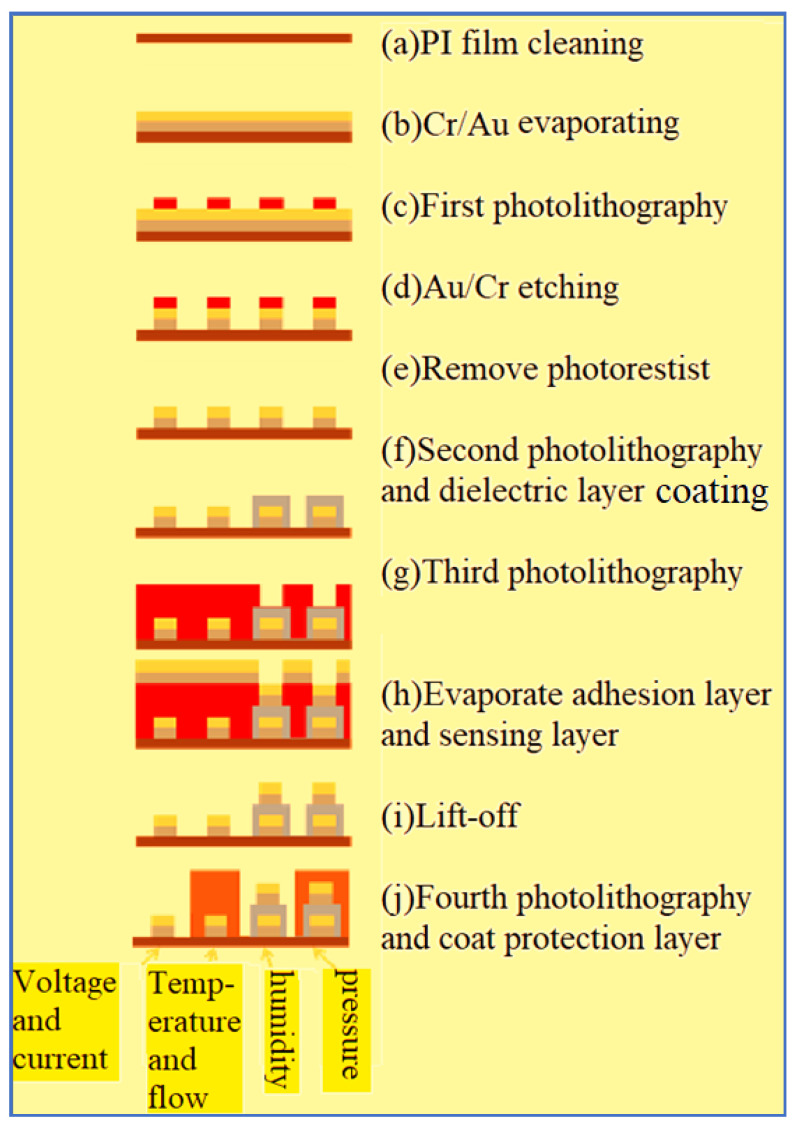
Process chart of flexible six-in-one microsensor.

The process is performed by using surface micromachining technology, including deposition, lithography, wet etching and metal lift-off. The fabrication process is shown in Figure 7, elaborated below.

(a) PI film cleaning and fixing

This study uses polyimide (PI) film as the substrate of the flexible six-in-one microsensor. The PI has good chemical resistance and mechanical strength, as well as insulation and a low thermal expansion coefficient compared with stainless-steel foil. The PI is soaked in organic solvent acetone to remove esters, and the residual acetone and dissolved impurities are cleaned off with methanol. Afterwards, it is cleaned by an ultrasonic oscillator for 3 min. Finally, the substrate is washed with deionized water to remove the residual methanol from the surface.

(b) Cr/Au evaporating

The Cr/Ti/Au is evaporated by an electron beam evaporator (EBS-500, Junsun technologies Co.). In terms of the principle of the electron beam evaporation method, the current is supplied to the tungsten filament to generate high heat, the kinetic energy of the outer electrons is larger than the binding energy, inducing spillage—known as thermal ionization. The high potential difference guides electron acceleration, the target material is focused through magnetic lens and the solid particle target material is heated to somewhere about melting point. When the evaporation material gets in a molten state, the material is evaporated and deposited on the upper substrate, forming thin film deposition. Table 1 shows the process parameters of evaporator.

(c) The first photolithography

The positive photoresist AZ^®^ P4620 is used for spin coating, soft bake, exposure and development.

(d) Au/Cr etching

When the pattern is transferred to the positive photoresist (AZ^®^ P4620), the pattern is transferred to the metal film of Cr and Au by using wet etching.

(e) Remove photoresist

After wet etching, the photoresist on the structure is removed by acetone and methanol.

(f) The second photolithography and dielectric layer coating

This study uses PI 9305 as the material of dielectric layer. Therefore, the purpose of the secondary exposure and development is to define and complete the dielectric layer of micro humidity sensor.

(g) The third photolithography

This study uses negative photoresist APOL-3202 as the sacrificial layer of the metal lift-off method. There will be a little expansion after photolithography, which will be helpful to subsequent evaporation process, because the evaporated metal cannot cover the photoresist completely, favorable for the photoresist to contact remover, guaranteeing the completion of metal lift-off method.

(h) Evaporate adhesion layer and sensing layer

The process steps are the same as (b).

(i) Lift-off method

The photoresist is soaked till the metal is completely lifted off.

(j) The fourth photolithography and coat protection layer

**Figure 8:**
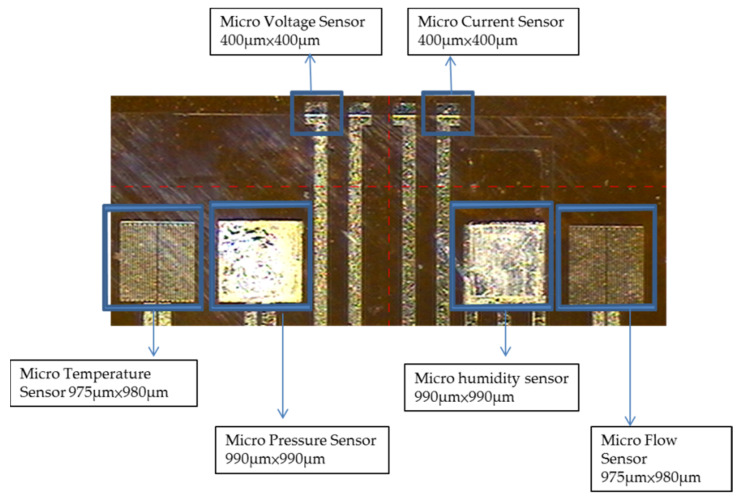
Optical micrograph of flexible six-in-one microsensor.

To avoid the flexible six-in-one microsensor being damaged by the closing pressure of the end plate inside the PEMWE, the insulation protection layer must have high mechanical strength and be adapted to the highly chemical environment. On the other hand, the protection layer can avoid the short circuit of the flexible six-in-one microsensor with a graphite plate. This study uses the PI9320 as the protection layer, the flexible six-in-one microsensor is fabricated by using photolithography again and coating protection layer. The optical microphotograph is shown in Figure 8.

## 4. Internal Real-Time Microscopic Diagnosis of PEMWE

After the fabrication of the flexible six-in-one microsensor, the six-in-one microsensor is encapsulated and corrected for convenient signal measurement and validating the reliability. Three flexible six-in-one microsensors are corrected one by one, after the reliability is confirmed, they can be embedded in the PEMWE for internal real-time microscopic diagnosis, so as to guarantee the correctness of experiment data.

### 4.1. Voltage Distribution inside Water Electrolyzer

**Figure 9:**
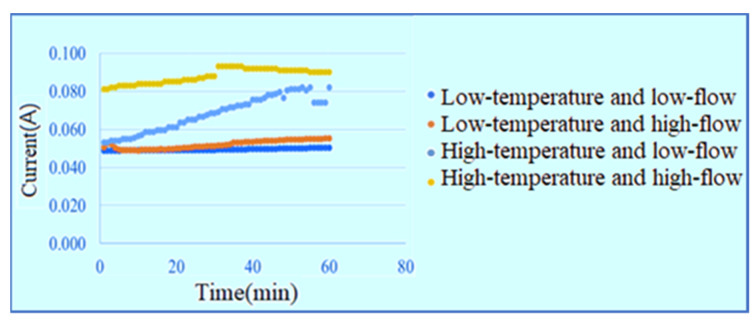
Current data of power supply.

**Figure 10:**
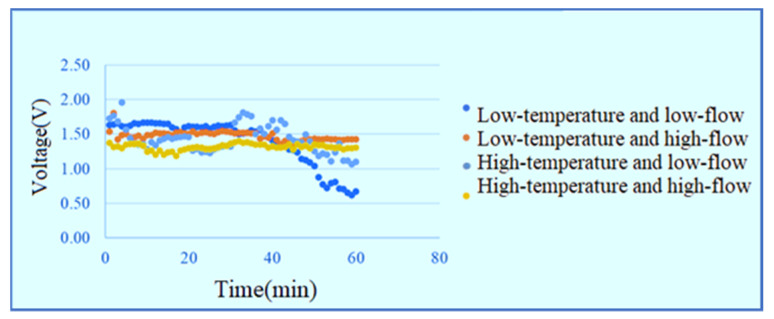
Voltage data at lower inlet of runner.

**Figure 11:**
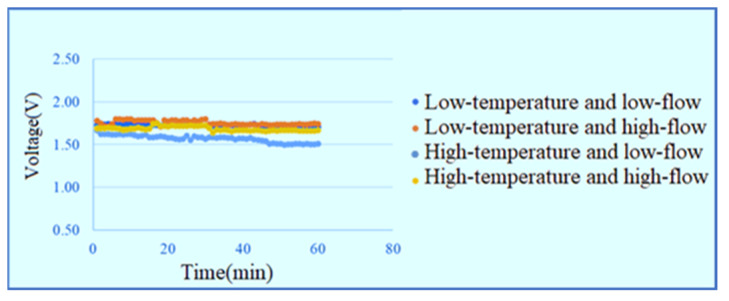
Voltage data at upper outlet of runner.

The temperatures of the DI water admitted into the PEMWE are 25 and 60 °C and the flow rates are 30 and 100 mL/min, respectively. The measurement is performed with a constant voltage of 1.8 V in the low-temperature low-flow, low-temperature high-flow, high-temperature low-flow and high-temperature high-flow conditions for one hour. The signals are captured once per minute and the current data of the power supply shows that the high-temperature high-flow has the best efficiency, as shown in Figure 9. The inlet voltage data are shown in Figure 10. The outlet voltage data are shown in Figure 11. It is observed that the voltage at lower inlet changes drastically in the reaction process.

### 4.2. Current Distribution inside Water Electrolyzer

**Figure 12:**
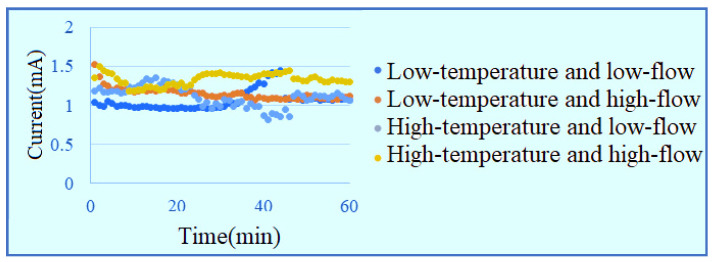
Current data at lower inlet of runner.

**Figure 13:**
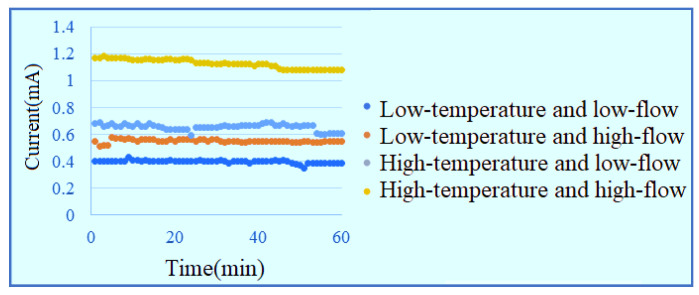
Current data at upper outlet of runner.

The measurement is performed with a constant voltage of 1.8 V in low-temperature low-flow, low-temperature high-flow, high-temperature low-flow and high-temperature high-flow conditions for one hour. The signals are captured once per minute. The inlet current data are shown in Figure 12. The outlet current data are shown in Figure 13. It is observed that the current at lower inlet changes drastically in the reaction process, and the current at outlet with high flow is obviously higher than the current data of low flow.

### 4.3. Temperature Distribution inside Water Electrolyzer

**Figure 14:**
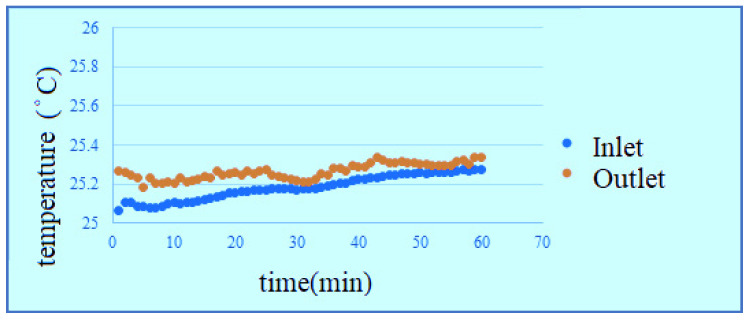
Low-temperature low-flow outlet/inlet temperature curve diagram.

**Figure 15:**
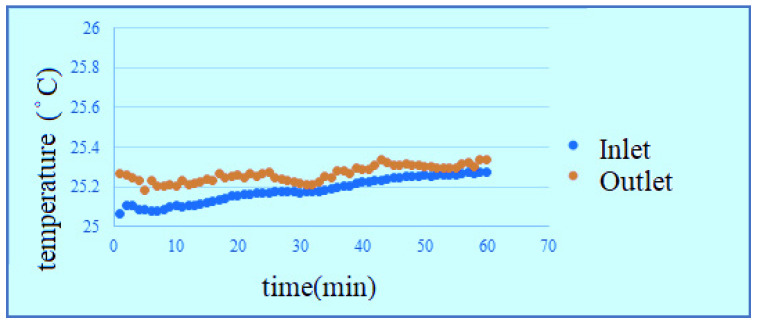
Low-temperature high-flow outlet/inlet temperature curve diagram.

**Figure 16:**
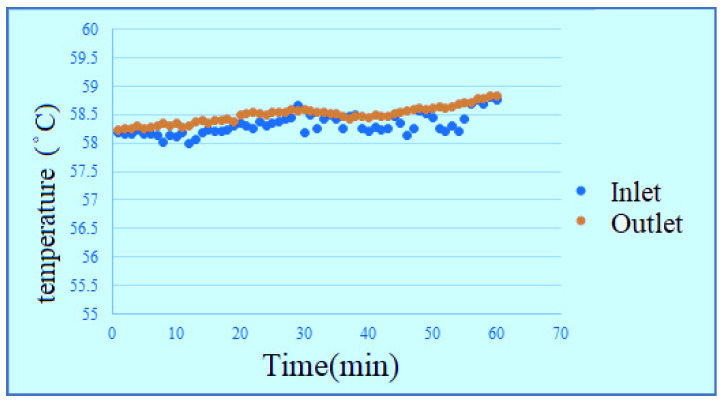
High-temperature low-flow outlet/inlet temperature curve diagram.

**Figure 17:**
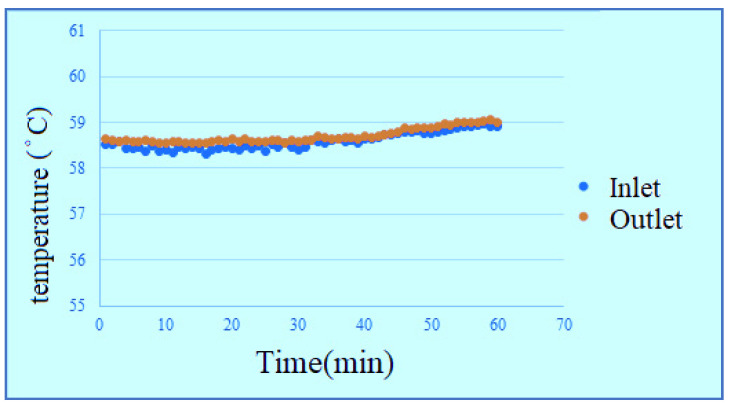
High-temperature high-flow outlet/inlet temperature curve diagram.

According to the data, the change of temperature at the inlet is more obvious in the reaction process, the outlet temperature changes smoothly, but the temperature is universally high, because the water is filled into the PEMWE through the lower inlet, the water generates a little heat after reaction, the temperature rises. The temperature at upper outlet rises slightly as the water is transferred. According to the figures, the effect of waste heat is not obvious in hot environment, and the temperature change of high flow is relatively smooth, as shown in Figure 14, Figure 15, Figure 16 and Figure 17. It can be seen from the figure that the temperature is very gentle when the flow is high, and the temperature is more unstable when the flow is low, and the figure shows that the temperature will not have much effect.

### 4.4. Humidity Distribution inside Water Electrolyzer

**Figure 18:**
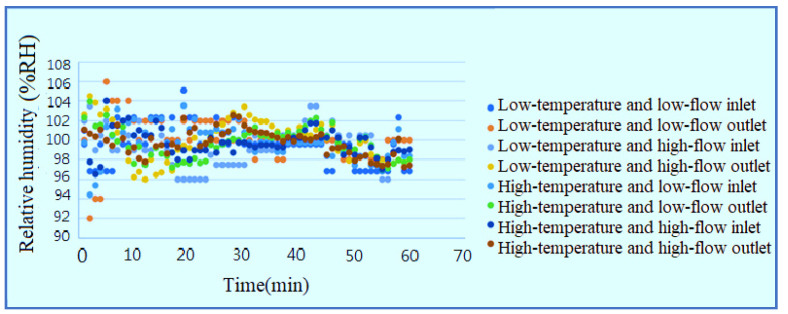
Outlet/inlet humidity curve diagram.

According to the data, the humidity at the outlet/inlet is 100% in the reaction process, the resistive micro humidity sensor has ±10% error due to temperature effect, as shown in Figure 18. Because it is measured under different conditions, the conversion of the environment causes errors in the measurement of the measurement equipment.

### 4.5. Flow Distribution Inside Water Electrolyzer

**Figure 19:**
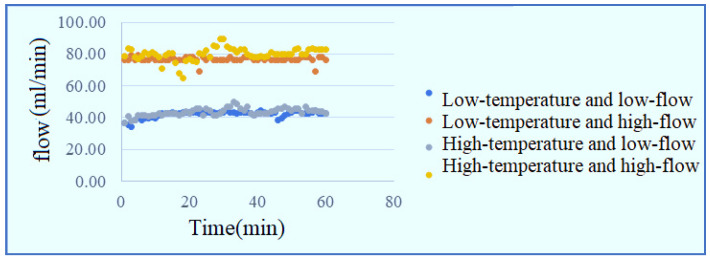
Curve diagram of flow at lower inlet of runner.

**Figure 20:**
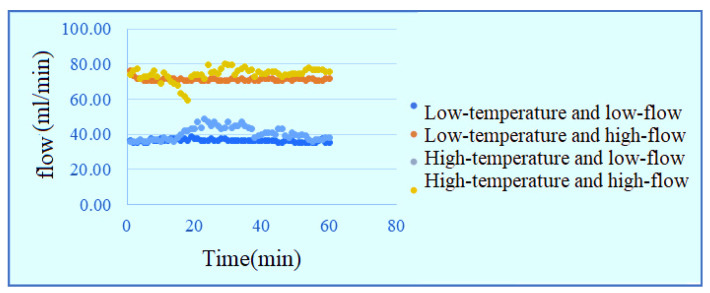
Curve diagram of flow at upper outlet of runner.

Figure 19 and Figure 20 show the local flow distribution inside PEMWE. It is observed that the flow at the runner inlet is faster, that at the runner outlet is slower, it may because the fluid of the runner design is smooth and stable in comparison to snakelike runner.

## 5. Conclusions

This study uses MEMS technology to develop a flexible six-in-one microsensor resistant to the electrochemical environment, the micro voltage, current, temperature, humidity, flow and pressure sensors are integrated on a 50 μm thick PI film substrate successfully, and the PI (Fujifilm Durimide^®^ PI 9320) resistant to the corrosion of electrochemical environment is used as protection layer. This flexible six-in-one microsensor has six functions and many advantages, such as corrosion resistance, small volume, high sensitivity, good temperature tolerance, real-time measurement and arbitrary placement. The flexible six-in-one microsensor successfully extracts local voltage, current, temperature, humidity and flow information inside the PEMWE without influencing the operation of PEMWE.

## Figures and Tables

**Table 1:** The process parameters of evaporator.

Evaporator rate	0.1 Å/s
Evaporator thickness	1000 Å
